# The effect of respiratory motion compensation in intracardiac 4D flow magnetic resonance imaging on left ventricular flow dynamics, multicomponent particle tracing, and valve tracking

**DOI:** 10.1093/ehjimp/qyaf020

**Published:** 2025-03-10

**Authors:** Paul R Roos, Thomas in de Braekt, Hildo J Lamb, Jos J M Westenberg

**Affiliations:** Department of Radiology, Leiden University Medical Center, P.O. Box 9600, 2300 RC Leiden, The Netherlands; Department of Radiology, Leiden University Medical Center, P.O. Box 9600, 2300 RC Leiden, The Netherlands; Department of Radiology, Catherina Hospital, Eindhoven, The Netherlands; Department of Radiology, Leiden University Medical Center, P.O. Box 9600, 2300 RC Leiden, The Netherlands; Department of Radiology, Leiden University Medical Center, P.O. Box 9600, 2300 RC Leiden, The Netherlands

**Keywords:** 4D flow, intracardiac, flow dynamics, particle tracing, valve tracking

## Abstract

**Aims:**

4D flow magnetic resonance imaging (MRI) has enabled evaluation of intracardiac flow dynamics by particle tracing for visualizing and quantifying complex flow patterns. The aim of this study was to assess the impact of respiratory motion compensation on 4D flow MRI–based left ventricular four-component particle tracing, valve tracking, and haemodynamics.

**Methods and results:**

In this prospective cohort study, 4D flow MRI with and without respiratory motion compensation was performed in 15 healthy volunteers. Intracardiac particle tracing considered four components: direct flow, delayed ejection flow (DEF), retained inflow (RI), and residual volume. Data quality was assessed by comparing DEF and RI components. Particle tracing, valve tracking, kinetic energy (KE), and vorticity were compared between scan methods. Paired sample *t*-tests and intraclass correlation analysis were performed with an alpha of 0.05. DEF, RI, ejection fraction, and stroke volume were different between scan methods. Five participants showed DEF-RI mismatch > 10%. After excluding these, differences in flow fractions were non-significant. Differences in stroke volume, ejection fraction, and valvular flow mismatch between scan methods remained. Valve tracking was comparable between scan methods and correlated well with particle tracing. Absolute mismatch between particle tracing– and valve tracking–based mitral flow, and KE and vorticity at A-peak, was higher for non-compensated MRI.

**Conclusion:**

Respiratory motion compensation can improve accuracy of intracardiac particle tracing based on 4D flow MRI by decreasing mismatch to retrospective valve tracking. For intracardiac particle tracing, respiratory motion compensation is advised. Robust data quality assessment for particle tracing–based analyses is equally crucial.

## Graphical Abstract

**Graphical Abstract:**
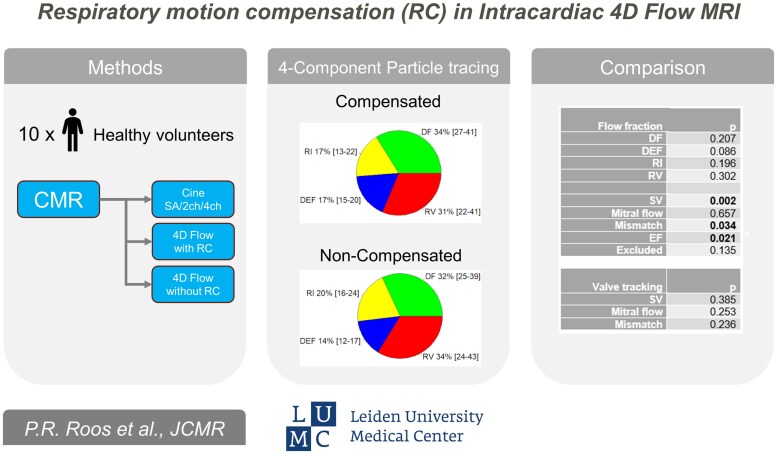

## Introduction

Cardiovascular magnetic resonance (CMR) imaging is a non-invasive imaging technique that provides unique insights into cardiovascular function and structure.^1^ It has become an almost vital part in diagnosis and follow-up of conditions such as congenital heart disease, cardiomyopathies, and valvular heart disease. Time-derived 3D three-direction phase contrast MRI, also called 4D flow MRI, is a CMR technique that measures the velocities of blood flow throughout the cardiac cycle.^2,3^ The ability to obtain both spatial and temporal information regarding blood flow has paved the way for applications such as intracardiac particle tracing, enabling the visualization and quantification of complex flow patterns in the heart chambers.^4,5^ This technique involves releasing virtual particles into the 4D flow data and calculating their trajectory throughout the cardiac cycle.^6^ In this way, the flow distribution inside the cardiac chambers can be described by four flow components [i.e. direct flow (DF), delayed ejection flow (DEF), retained inflow (RI), and residual volume (RV)] that reflect the function of the heart. However, the accuracy and reliability of particle tracing depend not only on the spatial and temporal resolution of the imaging data but also on the compensation of respiratory motion, which can introduce significant artefacts and errors into the analysis.^7^

Intracardiac particle tracing can give an extensive evaluation of cardiac function, but its applicability in routine clinical use is limited by the long scanning times of the 4D flow MRI and the complexity of the post-processing.^4^ The acquisition times typically range between 10 and 20 min and are limited by the vast amount of data that needs to be scanned, as well as the use of respiratory motion compensation.^3^ The latter is generally performed using retrospective motion compensation (i.e. the motion of the hemidiaphragm), in which data acquired outside a predefined respiratory motion window are disregarded, or prospective motion compensation, in which data are only acquired for a short period after a specific part of the respiratory cycle has been reached.^3^ Both methods substantially increase the acquisition time.^3^ Furthermore, common respiratory motion compensation techniques may carry inherent disadvantages. Navigator beam motion compensation can limit the temporal resolution and may cause artefacts by interrupting the acquisition, which may influence the steady state and result in artefacts.^8,9^

Due to its anatomical position, the heart moves with the respiratory motion throughout the respiratory cycle. While this motion might only have a limited effect in some post-processing analyses such as valvular flow analysis, it is larger than the blood volume represented by a particle in particle tracing.^10,11^ Additionally, inaccuracies and noise in the data are intensified by the discrete integration performed in particle tracing.^11^ Previous studies into the need and methods of respiratory motion compensation in 4D flow MRI have only focused on the thoracic aorta and other haemodynamic parameters than particle tracing.^12,13^ In this paper, we aim to quantitatively and qualitatively assess the effect of respiratory compensation on 4D flow–based intracardiac four-component particle tracing, valve tracking, kinetic energy (KE), and vorticity in the left ventricle (LV) and thereby provide insight into the role of respiratory motion compensation in cardiac 4D flow MRI.

## Methods

### Acquisition

**Figure 1:**
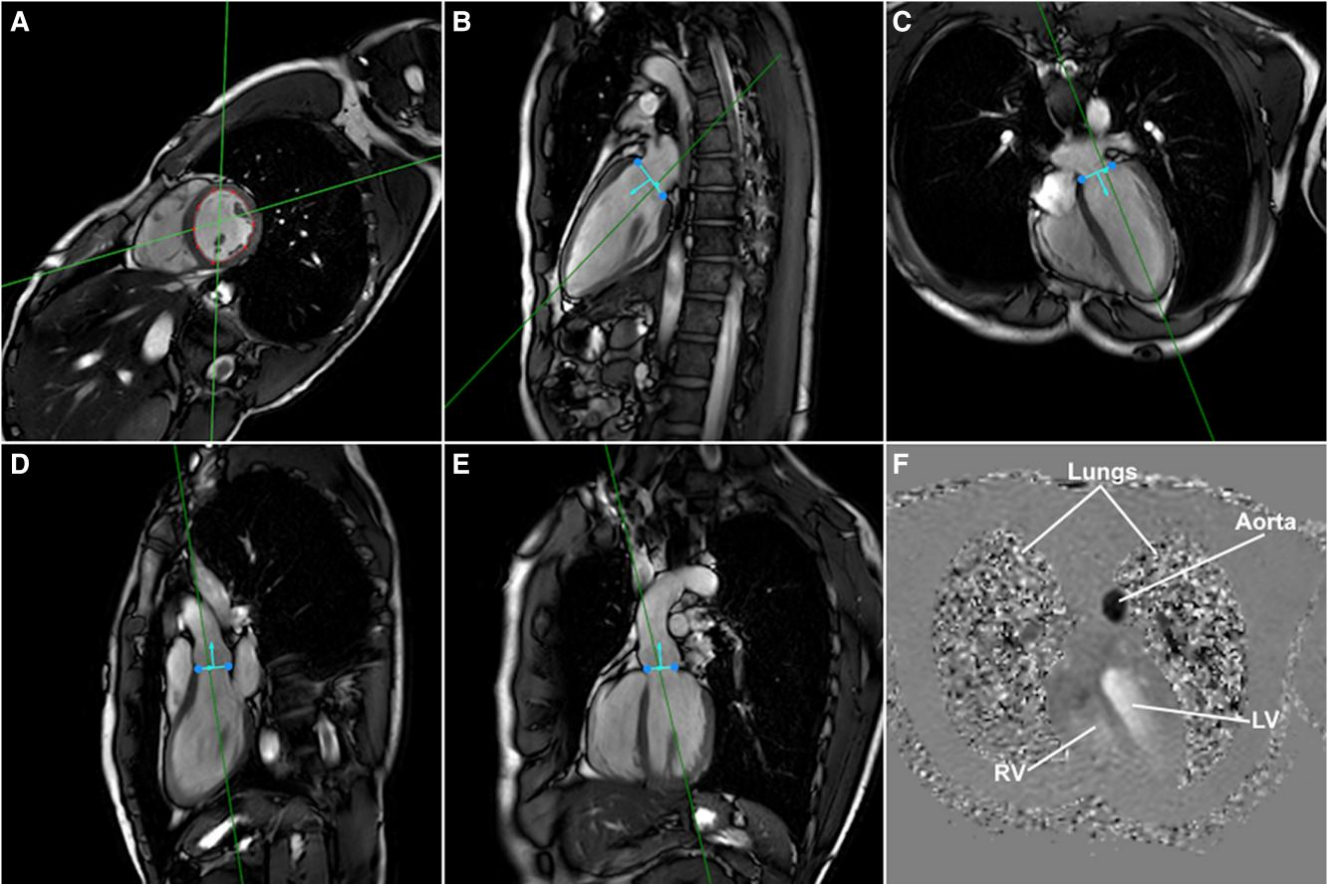
CMR scans with segmentation and valve tracking, as used to perform four-component intracardiac particle tracing. (*A–E*) LV cine segmented fast gradient echo scans of short-axis, two-chamber left, four-chamber, sagittal aortic annulus, and coronal aortic annulus, respectively. Straight lines depict location and orientation of scans that are perpendicular to the image plane. Blue lines and arrows depict mitral annulus (*B* and *C*) or aortic annulus (*D* and *E*) location and flow direction. (*F*) Phase image of 4D flow MRI with velocity encoding in feet–head direction with time point chosen during peak-systole.

Cardiovascular magnetic resonance (CMR) imaging was performed in 15 (*n* = 15) healthy volunteers [mean age 27 ± 8years, 11 male (73%), *Table 1*] with a Philips Ingenia Elition X 3.0T CMR scanner (Philips Healthcare, Best, The Netherlands). The comprehensive scan protocol encompassed a short-axis cine segmented fast gradient echo (SSFP) scan, along with two-chamber and four-chamber cine scans (*Figure 1A–C*). These scans were performed during a breath-hold at end-expiration. Additionally, two perpendicular cine scans were acquired through the aortic outflow tract, supplemented by two cardiac 4D flow MRI scans (*Figure 1–F*). These 4D flow scans encompassed the entire heart and had an encoding velocity (Venc) of 150 cm/s in all spatial directions. The first 4D flow scan incorporated retrospective respiratory motion compensation through navigator gating at end-expiration utilizing weighted gating with a starting window of 6 mm, which was extended to 25 mm after 25% of the data was acquired. The second 4D flow scan lacked respiratory compensation (non-compensated). All scans were performed under the guidance of retrospective electrocardiogram gating. Other scan parameters are noted in *Table 2*. No specific dietary or equivalent restrictions were imposed upon the participants during the imaging sessions.

**Table 1:** Healthy volunteer demographics

	Healthy volunteers
*n*	15
Male	11 (73%)
Age (years) (std.)	27 (±8)

**Table 2:** Scan parameters for cine scans and 4D flow MRI

	Cine 2ch/4ch/Ao	Cine short-axis	4D flow
FoV (mm^3^)	350 × 350 × 8.0	350 × 350 × 104^a^	370 × 395 × 104^a^
Acquired resolution (mm^3^)	2.0 × 1.6 × 8.0	1.5 × 1.5 × 8.0	3.5 × 3.5 × 3.5
Reconstructed resolution (mm^3^)	1.0 × 1.0 × 8.0	0.73 × 0.73 × 8.0	2.0 × 2.0 × 1.75
Slice gap (mm)	0	0	−1.5
TR (ms)	2.6	3.0	5.1
TE (ms)	1.3	1.5	3.0
Flip angle (°)	45	45	5
Sense factor	2	2.5	3/1.4
Segmentation factor	17–18	16	2
Respiratory compensation	Breath-hold	Breath-hold per slice	Navigator gating or none
Cardiac phases^b^	30	30	30
Venc (cm/s)			150

^a^Average field of view (FoV) as the scan encompassed the entire heart.

^b^Across all scan types, 30 cardiac phases were reconstructed, unless constrained by the prevailing heart rate of the respective volunteers at the time of scanning.

The study has been performed in accordance with the ethical standards in the 1964 Declaration of Helsinki, and all study participants gave informed consent prior to scanning, and the study was approved by the Medical Ethics Committee Leiden The Hague Delft.

### Image analysis

The delineation of epicardial contours was conducted on the cine short-axis scan at the end-diastolic and end-systolic phase using CAAS MR Solutions V5.1 (PIE Medical Imaging, Maastricht, The Netherlands). The segmentation process was executed by a CMR researcher (P.R.R.) possessing a professional background of over 3 years in the field. The derived contours were subsequently exported, yielding LV models intended for employment in the ensuing particle tracing procedures.

Semi-automated valve tracking was conducted through the utilization of CAAS MR Solutions V5.2.2. Explicitly, the mitral and aortic annuli were demarcated on the two-chamber and four-chamber scans (pertaining to the mitral valve) as well as the orthogonal aorta scans (pertaining to the aortic valve) (*Figure 1B* and *C*). These identified landmarks were then subjected to an automated tracking procedure across all phases of the cardiac cycle, with manual corrections being incorporated when deemed necessary. After this process, reformatted through-plane velocity images were exported from the localized valve positions. Next, valve net forward flow was measured through manual contouring of the valve annulus. Phase background and anti-aliasing corrections were applied in CAAS MR Solutions.

Considering the potential misalignment between the acquired cine scans and the 4D flow scans, a process of image registration was executed. This alignment was achieved through the application of a rigid 3D transformation facilitated by the open-source toolkit SimpleITK.^14^ The transformation parameters thus derived were subsequently applied to the volumetric 3D models and the delineated valve planes, ensuring a congruent spatial alignment between the different imaging modalities.

### Particle tracing

Virtual massless particles were initiated at the central position of each voxel encompassed by the LV models within the 4D flow scans, maintaining a 2.5 mm margin from the LV wall to account for potential inaccuracies stemming from segmentation procedures. Commencing at the end-diastolic phase, the particles were propagated both forwards and backwards in time until reaching the end-systolic phase. The computation of particle trajectories was executed utilizing a fourth-order Runge–Kutta integration technique with a time step of 10 ms, as implemented in the publicly available SciPy package.^15^ The tracing trajectory was prematurely halted in instances where a particle intersected a valve plane or exceeded a displacement threshold of 3 cm from the end-diastolic LV model. Particles that exceeded 5 mm from the end-systolic LV model and remained unaffected by any valve planes were excluded from the post-processing analysis conducted at end-systole. The fraction of excluded particles was calculated.

After forward and backward temporal tracing, the particles were classified into four components based on their sequential origin and termination points throughout a complete cardiac cycle: *DF*, particles that ingress into the LV via the MV and subsequently egress through the AV; *DEF*, particles resident within the LV at commencement and departing through the AV during ejection; *RI*, particles that introduce into the LV via the MV and maintain their presence therein; and *RV*, particles already positioned within the LV at the outset and persisting therein.^4^ The total number of particles in the DF and DEF fractions was multiplied with the voxel dimensions to calculate LV systolic stroke volume (SV), while the DF and RI fractions were used to calculate the LV diastolic inflow volume through the mitral valve similarly.

Data quality was ascertained through a comparison of the DEF and RI components [Eq. (1)], recognized to approximate parity within a normally functioning heart. Additionally, the proportion of particles excluded from the analytical processes was quantified, serving as an ancillary metric for the overall quality of the methodologies employed. This excluded particles fraction is aimed to be lower than 20%.^11^


(1) Mismatch=DEF−RI

### Haemodynamics

KE and vorticity were calculated in all voxels within the endocardial contours of the LV at peak-systole, E-peak, and A-peak.^16^ These cardiac phases were identified from the valve tracking results. Total KE and total vorticity were calculated by summing results from all voxels inside the LV within the specific cardiac phase. Results were indexed against end-diastolic volume.

### Statistical analysis

Flow component magnitudes, net forward transmitral volume, indexed KE, and vorticity were all subjected to comparative analysis between the respiratory-compensated and non-compensated scans through the utilization of paired *t*-tests. Additionally, paired *t*-tests were employed to ascertain statistical distinctions in the proportions of excluded particles and the absolute disparities observed between the DEF and RI components. The difference between particle tracing– and valve tracking–based SV and mitral flow was calculated and assessed with paired *t*-tests.

## Results

### Four-component analysis and valve tracking

4D flow MRI acquisition time was estimated to have been around 15 min with respiratory motion compensation and around 8 min without, depending on number of slices, heart rate, and navigator efficiency. Average semi-automatic LV endocardial contour segmentation time was around 15 min, while average mitral and aortic valve tracking was around 10 min per CMR data set. An average of 17 500 (±4800) particles were seeded in all 30 analyses, which is equal to the number of voxels within the segmentation models. Total computation time was around 2.5 h for each analysis, depending on the number of particles. The mean fraction of excluded particles was 15.7% (SD = 10.4%), which is lower than the aimed threshold of 20%. This fraction was equally high between respiratory motion-compensated and non-compensated scan (*P* = 0.98).

**Figure 2:**
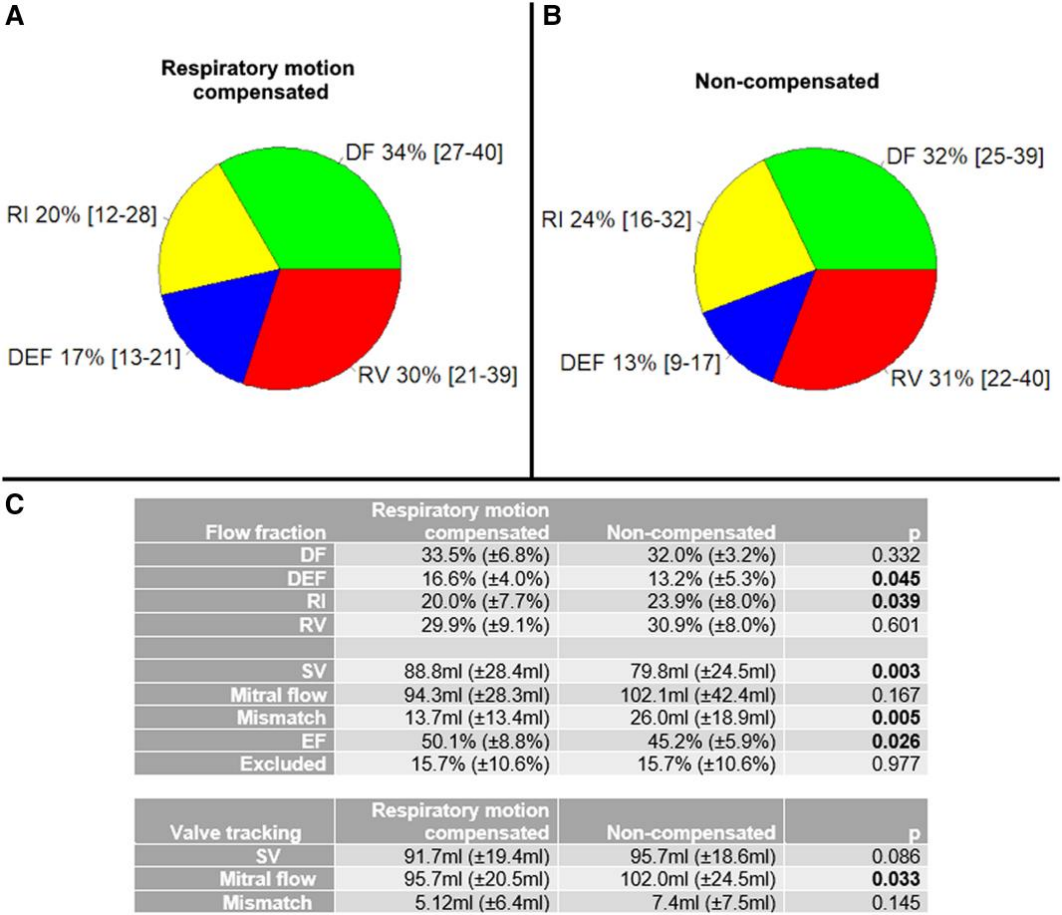
Intracardiac particle tracing flow components in 4D flow MRI with respiratory compensation (*A*) and non-compensated (*B*) in 15 healthy volunteers. Flow fractions and EF were compared with paired sample *t*-test (*C*), but no significant *P*-values were found. DF, direct flow; DEF, delayed ejection flow; RI, retained inflow; RV, residual volume; SV, stroke volume; Mismatch, |mitral flow–SV|.

DEF was significantly lower and RI significantly higher in the non-compensated compared with respiratory-compensated scans (*Figure 2*), as compared with paired sample *t*-test. Additionally, particle tracing–based SV was significantly lower in the non-compensated scans, as well as ejection fraction (EF). Mismatch between particle tracing–based mitral flow and SV was significantly larger in non-compensated scans, but mitral flow did not differ significantly. Differences in DF, RV, particle tracing–based mitral flow, valve tracking–based SV, and mismatch were not statistically significant.

There was good agreement between particle tracing– and valve tracking–based SVs (ICC = 0.71) and mitral flows (ICC = 0.76) overall, which was statistically significant. Overall particle tracing–based SV was significantly lower for non-compensated scans (mean difference = 15.9 mL), but not for motion-compensated MRI (*P* = 0.471). Mitral flow was not significantly different between methods for motion-compensated (*P* = 0.745) and non-compensated scans (*P* = 0.994). Mismatch between mitral flow and SV was significantly higher in particle tracing than in valve tracking for only non-compensated scans (mean difference = 18.5 mL).

### Visual analysis

**Figure 3:**
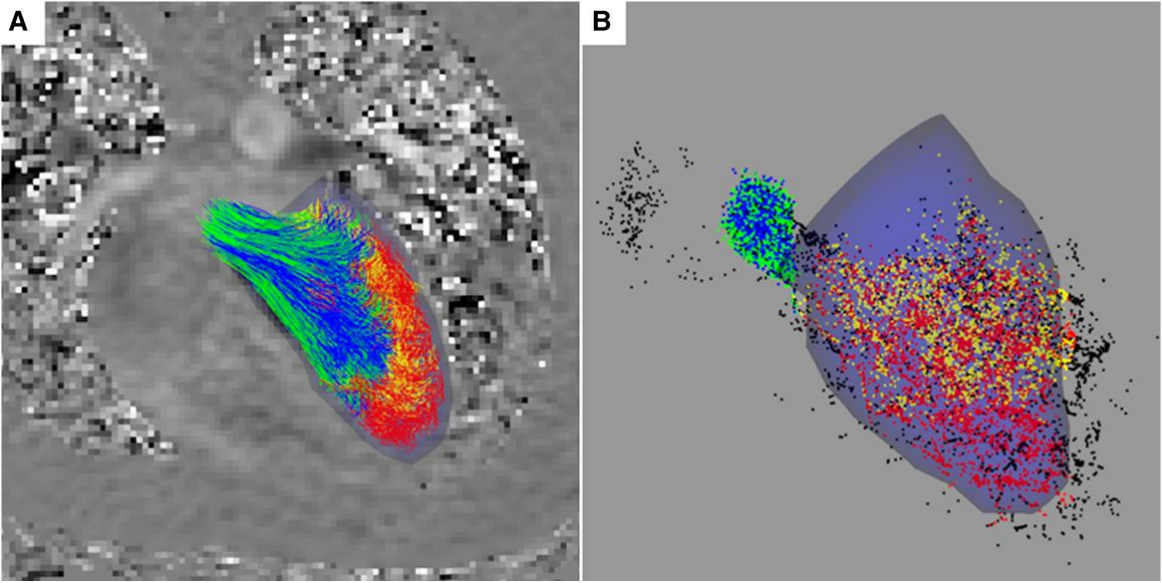
Example of visual results of four-component intracardiac particle tracing. Blue segmentation model of end-diastolic endocardial contours is drawn. Particles and pathlines are colour coded by flow component. (*A*) Pathlines from release to early systole, juxtaposed against 4D flow MRI slice (phase contract with feet–head flow encoding). (*B*) Particles at end of systole after forward particle tracing. Particles are black if excluded in either forward or backward particle tracing. Green, direct flow; blue, delayed ejection flow; yellow, retained inflow; red, residual volume; black, excluded particles.

Overall, the visual outcomes derived from the intracardiac particle tracing were clear and devoid of significant anomalies (example in *Figure 3*). We observed that in specific participants, the proximity of the major blood vessels in juxtaposition with the LV was high, resulting in instances where particles were erroneously directed out of the LV through these adjacent vessels, consequently warranting their exclusion from the subsequent analysis.

### Quality and flow mismatch

The fraction of particles that were excluded from analysis did not exhibit a statistically significant difference between respiratory motion compensation and non-compensated MRI (*P* = 0.97), as evidenced by an average of 15.7% and 15.7%, respectively (*Figure 2C*). The absolute mismatch between DEF and RI fractions was on average 7.8% (SD = 7.3%) for respiratory motion-compensated and 13.2% (SD = 7.3%) for non-compensated MRI data, which was statistically significant (mean difference = 5.4%).

A subset of cases (*n* = 5) demonstrated an absolute mismatch surpassing 10% (mean 17.4%) for both scan methods. Visual analysis in these specific cases did not reveal any major anomalies, nor could a correlation between the mismatch and the fraction of excluded particles (*R* = −0.214, *P* = 0.26) be found. Subsequent retracing employing reduced time steps and increased margins adjoining the ventricular walls yielded no discernible differences in results. Excluding these five cases from the analysis for inaccuracies yielded a mean absolute mismatch of 6.4% (SD = 4.9%). The mismatch was still significantly higher in non-compensated scans (mean difference = 6.0%). The exclusion of these five cases with inaccurate data yielded the following results.

### Respiratory motion compensation vs. no compensation for the selected data

**Figure 4:**
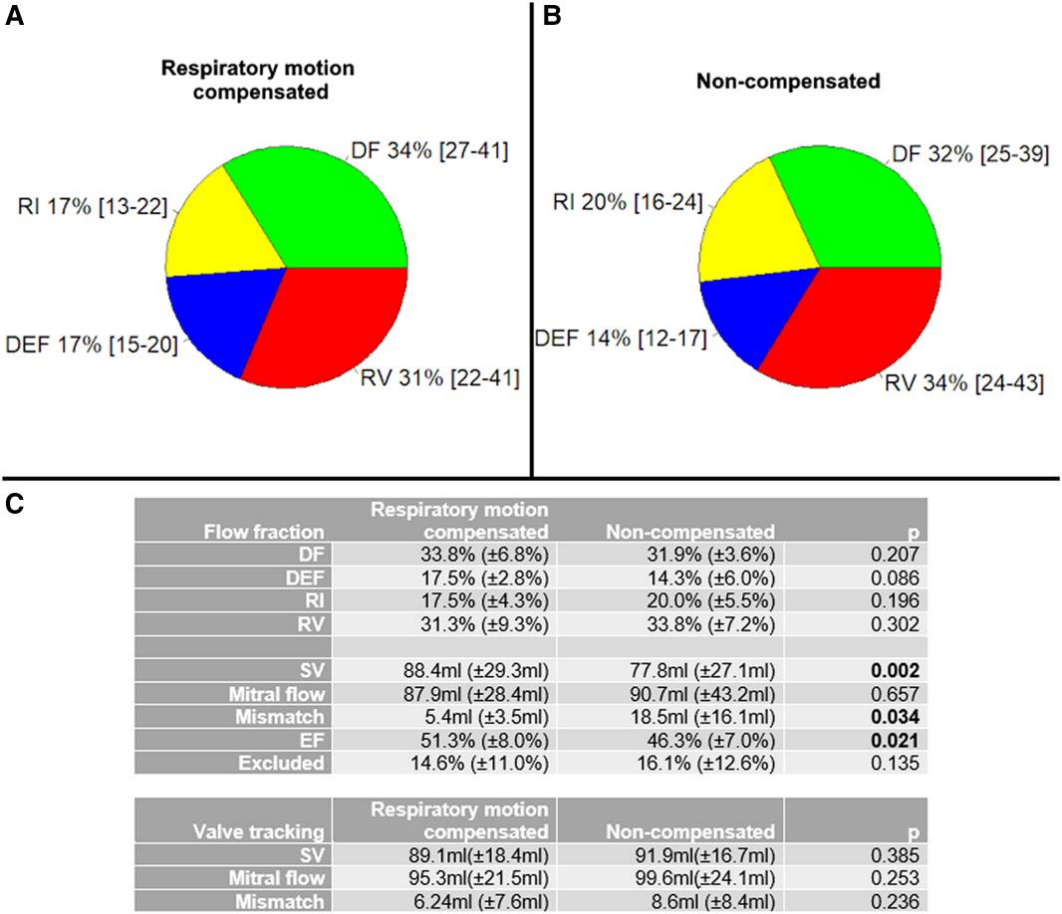
Intracardiac particle tracing flow components in 4D flow MRI with respiratory compensation (*A*) and non-compensated (*B*) in 10 healthy volunteers after exclusion of inaccurate data. Inaccurate particle tracing analyses were excluded from the data set. Flow fractions and EF were compared with paired *t*-test (*C*), but no significant *P*-values were found. DF, direct flow; DEF, delayed ejection flow; RI, retained inflow; RV, residual volume; SV, stroke volume; Mismatch, |mitral flow–SV|.

No significant changes in flow fractions, EF, and excluded particles fraction were found between respiratory motion-compensated and non-compensated MRI data (*n* = 10), as assessed by paired sample *t*-tests (*P* > 0.05) (*Figure 4C*).

Particle tracing–based SV was significantly lower (mean difference 10.6 mL) and mismatch significantly higher (mean difference 13.1 mL) for non-compensated vs. motion-compensated scans, but mitral flow was not significantly different (*P* = 0.657). Valve tracking–based mitral flow (*P* = 0.253) and SV (*P* = 0.385) were also not significantly different between scan methods.

There was a significant correlation between particle tracing– and valve tracking–based SV and mitral flows for respiratory-compensated MRI (ICC = 0.80 and ICC = 0.867, respectively) and for non-compensated MRI (ICC = 0.693 and ICC = 0.743, respectively). Particle tracing–based mitral flow was not statistically significantly different from valve tracking–based mitral flow for respiratory-compensated (*P* = 0.072) and non-compensated scans (*P* = 0.286). Differences between particle tracing– and valve tracking–based SV were insignificant for respiratory-compensated MRI (*P* = 0.895), but significant for non-compensated MRI (mean difference = 14.1 mL). The absolute mismatch between particle tracing– and valve tracking based mitral flow was significantly higher in non-compensated MRI vs. motion-compensated MRI (mean difference = 10.2 mL, respectively), but difference in SV measured with both techniques was not significantly different between scan methods (*P* = 0.369).

**Figure 5:**
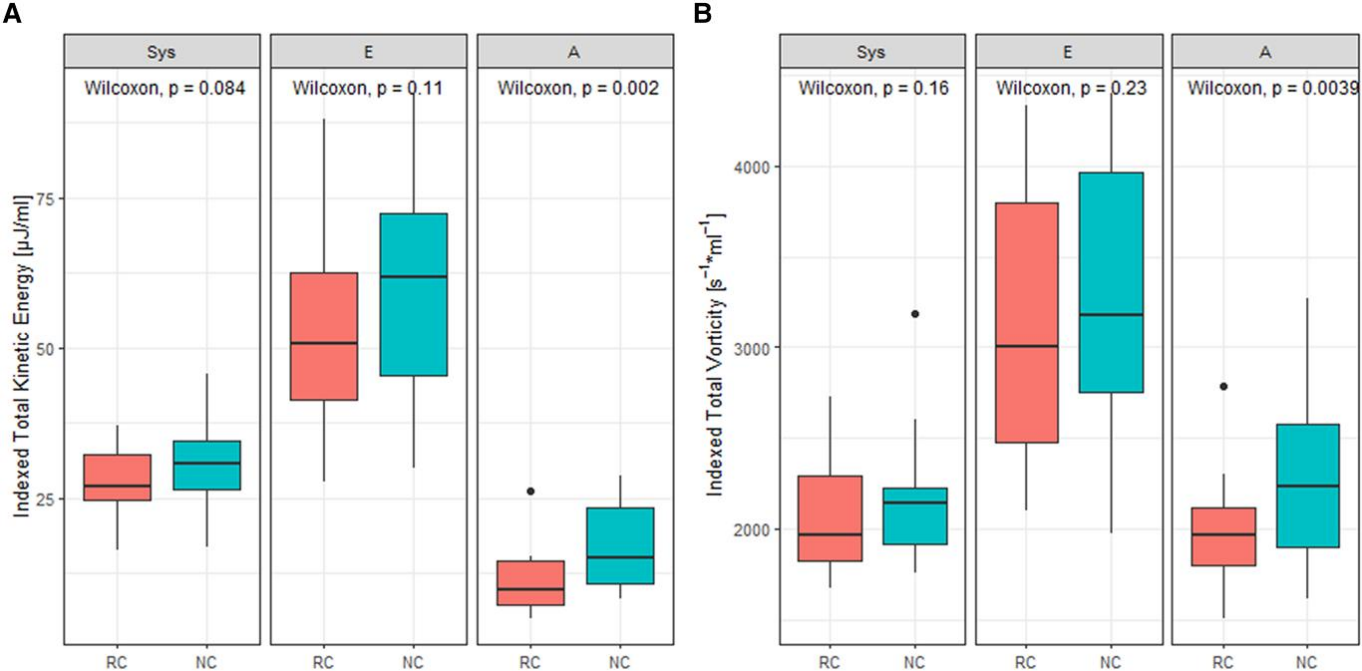
LV total KE (*A*) and total vorticity (*B*) at peak-systole (Sys), E-peak (E), and A-peak (A), indexed to end-diastolic volume. RC, respiratory motion-compensated MRI; NC, non-compensated MRI.

Total KE and total vorticity as assessed at peak-systole, E-peak, and A-peak were indexed by end-diastolic volume and are shown in *Figure 5*. Indexed vorticity correlated well between scan methods at peak-systole (ICC = 0.799), E-peak (ICC = 0.928), and A-peak (ICC = 0.726), which was also true for indexed KE at peak-systole and E-peak (ICC = 0.696 and 0.767, respectively). However, correlation between indexed KE with both scan methods was not significant at A-peak (*P* = 0.06). At the A-peak, indexed KE and vorticity were significantly higher in non-compensated scans than in respiratory-compensated scans. There were no significant differences at peak-systole and E-peak.

## Discussion

This study sought to investigate the influence of respiratory motion compensation on the process of four-component intracardiac particle tracing utilizing 4D flow MRI. We have shown significant differences in four-component analysis between respiratory motion-compensated and non-compensated 4D flow MRI, especially in DEF and RI, resulting in significant changes in EF, SV, and mismatch between SV and mitral flow. Additionally, significant differences between particle tracing– and valve tracking–based valvular flow were found in non-compensated MRI. This highlights the potential utility of respiratory motion compensation in enhancing the accuracy and reliability of these intracardiac analysis techniques.

Using respiratory motion compensation in cardiac 4D flow MRI is a key consideration, as it increases acquisition time significantly but may increase image quality and data accuracy.^12^ Whether this increase in accuracy is necessary for accurate analyses is not entirely clear and depends on other factors, such as the direction of the evaluated blood flow relative to the direction of the respiratory motion.^12^ The efficiency of respiratory motion compensation techniques on 2D flow MRI has been studied frequently, but only few studies investigated the effect on 4D flow MRI.^17–22^ These previous studies have assessed the effect of specific respiratory motion compensation on flow parameters such as net flow and flow rates in the major arteries and veins, particle tracing–derived volumes, or simulated phantom scans.^8,10,12,23^ Kanski *et al*.^10^ concluded from their study that cardiac 4D flow MRI can be acquired without respiratory gating with preserved quantitative results in particle tracing–derived volumes, KE, and vortex ring volumes, with Pearson correlation factors of at least *R*^2^ = 0.70. However, more advanced techniques such as advanced particle tracing analyses, intracardiac four-component analysis, and valve tracking had not been assessed yet. Accurate particle tracing relies on precise measurement, as the massless virtual particles follow the velocity vectors at a discrete grid that is defined by the voxel size. The respiratory motion of the heart is much larger than that, sometimes encompassing multiple centimetres. This may cause an overall blurring of the velocity data, resulting in a loss of detail that particle tracing may require. This is the first study on the effect of the time-efficient respiratory motion compensation technique weighted gating on advanced particle tracing techniques such as four-component analysis.

### Four-component analysis

The analysis of four flow components within the LV, comprising DF, DEF, RI, and RV, forms the cornerstone of our investigation. Our study findings, before removing outliers, align with prior studies performed in healthy volunteers.^4,5,24,25^ Notable in these studies are the averaged results, which might conceal outliers and inaccuracies that are possibly preventing the analysis method from being used accurately in individual cases. The same is true in our study: in 5 out of 15 participants, we found a substantial mismatch between DEF and RI, in both scan methods, which is not physiologically possible. In fact, similar inaccuracies were found in 2 of the 12 healthy volunteers in a previous study by Fredriksson *et al*.^26^ from 2011. In their study of the flow distribution in the right heart, two subjects presented an in- and outflow discrepancy of more than 10% and were excluded from subsequent analysis. Other literature mentions no statistics of excluded participants, but reported similar averaged results, which underlines the importance of our study findings.

### Visual analysis

No large visual anomalies could be observed in either the respiratory motion-compensated data or the non-compensated data. Visual results revealed that the proximity of major blood vessels to the LV might result in particles passing into these flows and therefore a higher number of excluded particles in some participants. However, there was no apparent direct reason for the large mismatch between DEF and RI in the five cases that were excluded from further analysis. A meager DEF or RI might be partly explained by a mismatch of the segmentation model and the 4D flow data, resulting in non-moving particles that were outside of the blood volume but not excluded. These particles were then part of the RV component.

While the previous hypothesis might not completely explain the mismatch between DEF and RI, the absence of major apparent anomalies or a correlation between the mismatch and the fraction of excluded particles did correspond with a different hypothesis: inaccuracies in the 4D flow data may have caused some particles to deviate from their actual path to follow the path of other particles, subsequently diminishing one flow component while increasing another. Because of the nature of particle tracing, any error or noise in the data can accumulate over time and have substantial impact due to the integration procedure. Unfortunately, this cannot always be precisely assessed by visual analysis, as analysing individual particles is not feasible and the errors or noise can be too subtle to identify.

### Respiratory motion compensation

We have shown differences in flow fractions between respiratory motion-compensated and non-compensated MRI data, but after exclusion of the five cases with data abnormalities (*n* = 10), the remaining differences were not statistically significant in this study. Non-compensated MRI had significantly higher absolute mismatch between DEF and RI fractions and particle tracing–based SV and EF was lower, while mismatch between SV and mitral flow was higher. Therefore, non-compensated MRI had on average less favourable results.

Particle tracing–based SV and mitral flow correlated well with valve tracking results (ICC = 0.72 and 0.77, respectively), but correlation was lower for non-compensated than in respiratory-compensated MRI. Correlation was therefore slightly lower than a previous study that reported Pearson correlation coefficients of 0.88–0.95 for mitral flow and 0.81–9.87 for SV.^27^ Particle tracing–based mitral flow was not significantly different from valve tracking mitral flow for both scan methods, which is comparable with a previous study.^27^ However, particle tracing–based SV was significantly lower in non-compensated MRI (14.1 mL). The differences between particle tracing and valve tracking mitral flow were significantly larger in non-compensated MRI (mean difference = 10.2 mL). This shows that, while differences in particle tracing four components are not significant, particle tracing results deviate substantially more from valve tracking results when no respiratory motion compensation is used.

There were high correlations (ICC ≥ 0.7) between KE and vorticity at peak-systole and E-peak, similar to the results achieved by Kanski *et al*.^10^ This preceding study, however, exclusively presents a comparison of mean KE and vorticity, thereby lacking a comparison per cardiac phase. This present study shows significantly increased KE and vorticity at A-peak in non-compensated scans, with no correlation between indexed KE at A-peak between both scan methods. Respiratory motion potentially contributed to the observed elevated measurements, which might impact the evaluation of the LV diastolic function.

Besides data inaccuracies and artefacts, flow differences might be caused due to the influence of respiration on cardiac flow. This influence is however not fully understood.^28,29^ With the emergence of 5D flow MRI, which enables 4D flow data evaluation at different parts of the respiratory cycle, these flow differences might be further investigated in the future.^29^ Current four-component particle tracing does not take respiratory motion into account, but with 5D flow MRI, four-component particle tracing might be performed at different parts of the respiratory cycle.

In this study, retrospective respiratory gating with a navigator echo to track diaphragmatic motion was used. This technique has a low efficiency, thereby increasing acquisition times of the 4D Flow sequences by about two-fold. Recent techniques, such as self-gating and motion correcting with butterfly navigators or radial readouts, might provide improved acquisition times, but they are not readily available yet.^30–32^ The present study and similar previous studies may help clinicians decide whether to use current respiratory motion compensation for their specific applications.

### Inherent inaccuracies

Our 4D flow MRI study data had inherent inaccuracies. Small inaccuracies might be limiting particle tracing and particle tracing–based analyses due to this technique’s character, as it is prone to cumulation of errors because of its stepwise integration.^11^ It is therefore crucial to assess the data quality of the 4D flow MRI data, before results of particle tracing–based analyses are accepted. This assessment can be performed with the particle tracing–based analyses themselves, such as the DEF-RI mismatch measurement we performed. Some of our data had an excluded particles fraction of over 20%, which is higher than the previously advised threshold.^11^ However, as no inaccuracies could be proven in the four component analysis nor in the visual analysis, the data were included. The DEF-RI mismatch of over 10% was deemed too inaccurate and the five cases that exhibited this in both scans were excluded from further analysis, which improved the overall results. We included the averaged results prior to this exclusion to illustrate the effects of including these inaccuracies and to compare this with existing literature. It remains important to be critical about performing particle tracing in potentially inaccurate 4D flow MRI, as results such as visual analysis may not always provide a clear measurement of the data quality.

Due to possible movement of the healthy volunteers in between scans, registration between the short-axis scans and 4D flow MRI scans was performed. This registration was evaluated for every case prior to further analysis and was deemed correct in every case. Inaccuracies in further analysis due to registration errors are therefore very unlikely.

### Clinical implications

The potential necessity of incorporating respiratory motion compensation in intracardiac CMR procedures should not be underestimated, particularly when dealing with individual patient data. The enhanced accuracy in particle tracing might offer valuable clinical insights into cardiac function, helping diagnose and manage cardiovascular diseases more effectively. We would recommend using respiratory motion compensation when assessing individual cases to ensure good accuracy of four-component analysis and valvular flow assessment. In clinical patients with irregular respiratory motion, the efficiency of respiratory gating may be lower than in this study, resulting in longer acquisition times. This has to be taken into account when assessing whether respiratory motion compensation is required.

### Limitations

This study was only performed on healthy volunteers, and no patients with cardiovascular disease were included. Data quality might be different in patients, especially in those with irregular cardiac or respiratory motion. Additionally, only CMR scanners from a single vendor were used and potential differences in data quality and analysis results between CMR scanner and post-processing software from different vendors could not be assessed. Additional inclusion of patients in this type of study is difficult, as it would require repeating long 4D flow MRI scans which would be difficult to justify in a clinical setting.

Data quality control was assessed based on only available literature, which was limited. The acceptance criterium of maximum 10% mismatch between DEF and RI flow fractions was based on a single study and our own choice.^26^ Data quality results could not be compared with other literature, as this is very minimally reported in other literature.

The two 4D flow MRI scans were performed in the same order for every volunteer, after about 30 min of other cardiac MRI scans. Even though these last two scans required no interaction from the volunteer, such as breathing instructions, fatigue could have played a factor in the results. However, this factor is believed to be very modest and no volunteer reported any fatigue after scanning.

### Conclusion

Respiratory motion compensation techniques can improve accuracy of four-component intracardiac particle tracing based on 4D flow MRI by decreasing mismatch to retrospective valve tracking. Indexed vorticity and KE at peak A-wave was higher in scans without motion compensation. For intracardiac particle tracing, respiratory motion compensation is advised. Additionally, robust data quality assessment for particle tracing–based analyses is equally crucial, especially in clinical assessment of individual patients.

## Data Availability

The data underlying this article will be shared on reasonable request to the corresponding author.

## Lead author biography

Paul Roos is a PhD candidate at the Leiden University Medical Center. His main focus is non-invasive cardiovascular MRI and the liver–heart axis. He has a degree in Technical Medicine from the Technical University of Delft, Leiden University Medical Center, and Erasmus Medical Center. His interests include advanced medical imaging, such as 4D flow MRI and MR spectroscopy, computational fluid dynamics, and post-processing techniques for quantification and visualization of flow.
